# The Impact and Correlation of Anxiety and Depression on Pressure Pain Threshold of Acupoints in Patients with Chronic Pelvic Inflammatory Disease

**DOI:** 10.1155/2023/3315090

**Published:** 2023-11-21

**Authors:** Chenxi Liao, Yu Tan, Kai Wang, Xin Wen, Xiang Hu, Yefang Huang, Ying Li

**Affiliations:** ^1^College of Acupuncture and Tuina, Chengdu University of Traditional Chinese Medicine, Chengdu, China; ^2^Sichuan Second Hospital of Traditional Chinese Medicine, Chengdu, China; ^3^Department of Gynecology, The Affiliated Hospital of Chengdu University of Traditional Chinese Medicine, Chengdu, China

## Abstract

**Background:**

Chronic pelvic inflammatory disease (CPID) is a clinically common gynecological disease. Patients experience chronic pelvic pain and often accompany with emotional dysfunction. However, the impact and correlation of anxiety and depression on pain sensitization is not completely known.

**Objective:**

To explore the differences and correlations among anxiety, depression, and pressure pain threshold (PPT) of acupoints in patients with CPID.

**Methods:**

One hundred and forty-seven patients with CPID were recruited. The Visual Analog Scale (VAS) and short-form McGill Pain Questionnaire (SF-MPQ) were used to assess pain. Self-Rating Anxiety Scale (SAS) and Self-Rating Depression Scale (SDS) were used to evaluate the emotional state of patients. The PPT of acupoints was collected using an electronic Von Frey by two licensed acupuncturists.

**Results:**

The CPID patients were divided into anxiety-depression group (group A) or nonanxiety-depression group (group B), according to the SAS and SDS scores. Finally, there were 73 patients in group A and 74 patients in group B. Group A had significantly higher SAS, SDS, VAS, and SF-MPQ scores than group B (*P* < 0.05). In addition, significant differences were observed in the PPTs of ST28 (R), ST29 (R), SP10 (R), SP9 (R), SP9 (L), ST36 (R), and LR3 (L) between the two groups (*P* < 0.05). No considerable differences in PPTs at the other acupoints were observed between the two groups. SAS scores showed a positive correlation with PPTs of ST29 (R), SP10 (R), SP9 (L), ST36 (R), and LR3 (L). No remarkable correlation was observed between the SDS scores and PPTs.

**Conclusion:**

Anxiety and depression can affect the PPT of some acupoints in CPID patients, which may provide a reference for acupoint selection for acupuncture treatment of CPID with emotional disorders. This trial is registered with ChiCTR2100052632.

## 1. Introduction

Pelvic inflammatory disease (PID) is an inflammatory disorder of the female upper genital tract (including endometritis, salpingitis, pelvic peritonitis, and tubo-ovarian abscess), with a wide range of clinical manifestations [1, 2]. The cardinal symptom of PID is the abrupt onset of lower abdominal or pelvic pain in a sexually active woman [3]. The patients are also with signs of pelvic organ tenderness, as indicated by cervical lifting pain, uterine compression tenderness, and adnexal tenderness on bimanual examination [1]. PID is mostly caused by sexually transmitted infection (STI), such as chlamydia trachomatis, gonorrhoeae, ureaplasma urealyticum, or bacterial vaginosis-associated microbes [4–6]. The burden of PID in the United States is high [7], where 4.4% of women will have had PID sometime in their lifetime [8]. About 33.6% of women aged 25–44 years have experienced at least one episode of PID in England [9]. When PID is not treated timely or correctly, it may develop into chronic pelvic inflammatory disease (CPID), which means longer course of disease (usually more than one month) [1] and is difficult to cure.

Most CPID patients have symptoms of chronic pelvic pain, because of the continuous effects of inflammation. A study demonstrated that chronic pelvic pain after an acute episode of PID was common and associated with decreased physical and mental health [10]. Psychological morbidity was higher in women with pelvic pain than that in pain-free controls [11]. Patients with CPID may simultaneously experience pain and emotional disorders (anxiety and depression), but it is unclear how they affect each other. On this basis, the correlation between psychosocial factors and CPID-related pain needs further investigation.

The pressure pain threshold (PPT) is a semiobjective method used to measure pain [12, 13], which can reflect the central and local peripheral sensitization. Studies showed that the PPT changed when the body was in pain [14, 15]. Traditional Chinese medicine believes that acupoints can reflect the state of disease, the degree of change in the PPT may objectively relate to the intensity of acupoint sensitization. Clinical studies have confirmed that the PPT of acupoints changes when patients experience pain [16–18]. However, few studies investigated whether CPID patients with emotional disorders and patients with normal emotion have difference in PPT. This study aimed to explore the differences and correlations between anxiety, depression, and PPT of acupoints in patients with CPID.

## 2. Methods

The CPID patients were recruited from the outpatient department of the Affiliated Hospital of Chengdu University of Traditional Chinese Medicine (CDUTCM) from March 2021 to September 2022. All patients were diagnosed by clinicians in the Gynecology Department of the Affiliated Hospital of CDUTCM.

The study was performed according to the principles of the Declaration of Helsinki, approved by the Ethics Committee of the Affiliated Hospital of CDUTCM (NO. 2021KL-014), and registered at the Clinical Trial Registry (registration number: ChiCTR2100052632). All the subjects signed the informed consent.

### 2.1. Participants

Patients were enrolled if they fulfilled the following criteria: (1) diagnosed with pelvic inflammatory disease [19, 20]; (2) had symptoms or signs of pelvic pain; (3) with a disease course more than 1 month [1]; (4) aged from 18 to 50 years; (5) married or unmarried with a history of sexual life; (6) did not take psychotropic drugs such as anxiety and depression; (7) did not participate in any other clinical trials; and (8) signed an informed consent form.

Patients with any of the following conditions were excluded: (1) if in critical condition at acute stage; (2) if pregnant or lactating; (3) if complicated by malignant tumors or serious diseases of heart, brain, liver, kidney, urinary, digestive, or reproductive system; and (4) if had unclear conscious and unable to cooperate with the investigator to complete the study.

### 2.2. Outcome Measurements

Baseline data consisted of age, body mass index (BMI), duration of disease, education, and resting blood pressure. The Self-Rating Anxiety Scale (SAS) [21] and Self-Rating Depression Scale (SDS) [22] were used to evaluate the emotional state of patients. The SAS Index score of 45 (raw score = 36) is a cut-off point for clinically significant anxiety [23], the SDS Index score of 50 (raw score = 40) suggests clinically significant symptoms of depression [24]. Higher scores indicate greater severity of anxiety and depression symptomology. The pelvic pain in this study referred to the pain during the onset of pain in the lower abdomen in patients and was measured by a horizontal Visual Analog Scale (VAS) ranging from 0 to 10, 0 referred to no pain, 10 referred to intense and unbearable pain, and higher score indicates more severe pain [25, 26]. The patient selected the corresponding number based on the degree of pain in their lower abdomen. Short-form McGill Pain Questionnaire (SF-MPQ) was also used, which provided valuable information on the sensory, affective, and overall intensity of pain [27].

PPT data (g) were collected using an electronic Von Frey (2390-type made by the IITC Company, United States) to apply pressure at a constant speed on acupoints. The acupuncturist held the detector and moved the probe tip vertically downward the skin at an even speed. The probe tip was immediately removed once the patients felt a sensation of pain and recorded the number on the detector as PPT [28]. All the acupoints were tested two times at an interval of five minutes. If the difference between the two PPT measurements was less than 15 g, the mean of these two times served as the final PPT of the acupoint. If there was a difference between the two PPT measurements of more than 15 g, the acupoint was measured for the third time. The average of the two values with the smallest difference was taken as the final PPT [29–31].

In accordance with the results of literature data-mining [32] and expert consensus on the acupuncture treatment of CPID, we identified 15 most frequently used acupoints as the test points (Table 1), included Daimai (GB26), Tianshu (ST25), Qihai (CV6), Guanyuan (CV4), Zhongji (CV3), Qugu (CV2), Shuidao (ST28), Guilai (ST29), Qichong (ST30), Zigong (EX-CA1), Xuehai (SP10), Yinglingquan (SP9), Zusanli (ST36), Sanyinjiao (SP6), and Taichong (LR3). All the acupoints were detected on both sides, except for CV6, CV4, CV3, and CV2.

### 2.3. Data Collection

The eligible patients completed the questionnaire survey and PPT test. Relevant data, including demographic information, SAS, SDS, VAS, and SF-MPQ scores, were independently collected by blinded evaluators. The PPT data were collected by another two blinded licensed acupuncturists.

### 2.4. Statistical Analysis

The SPSS version 26.0 software was used for statistical analysis by the blinded evaluator. Categorical data were described as number and percentages (*n*%) and compared using the *χ*^2^ or Fisher's exact test. Normally distributed quantitative variables using *t*-test were expressed as mean (*M*) ± standard deviation (SD). Skewed quantitative variables were analyzed by the Mann–Whitney *U* test, expressed as median (IQR). Spearman correlation coefficient was employed for nonnormal distribution data for correlation analysis. *P* value <0.05 was considered statistically significant.

## 3. Results

A total of 495 patients were screened, from which 348 did not meet the inclusion criteria and were excluded; therefore, 147 patients participated in the study. If the SAS score was greater than 45 or the SDS score was greater than 50, the patients were assigned to the anxiety-depression group (group A). Otherwise, if the SAS score was less than 45 and SDS score was less than 50, the patients were assigned to nonanxiety-depression group (group B). Finally, there were 73 patients in group A and 74 patients in group B.

### 3.1. Baseline Characteristics

The baseline data included age, BMI, education level, resting blood pressure, and duration of illness. Except for duration of illness, no remarkable differences in baseline data were found between the two groups (*P* > 0.05; Table 2).

### 3.2. The Emotion and Pain Scores in the Two Groups

Compared with group B, the group A had significantly higher SAS scores (median 49 (IQR, 45–54) vs. 38.5 (IQR, 34–41); *Z* = −9.151, *P* < 0.05) and SDS scores (median 53 (IQR, 46–59) vs. 40 (IQR, 35–43); *Z* = −7.721, *P* < 0.05). Significant difference in VAS scores (5.15 ± 1.82 vs. 4.20 ± 1.93; *t* = 3.083, *P* < 0.05) was found between the two groups. In addition, the SF-MPQ scores in the group A were also greater than group B (median 14.3 (IQR, 10.7–19) vs. 10.25 (IQR, 8.38–14.70); *Z* = −3.960, *P* < 0.05; Table 3).

### 3.3. The PPTs in the Two Groups

The PPTs of ST28 (R), ST29 (R), SP10 (R), SP9 (R), SP9 (L), ST36 (R), and LR3 (L) in group A were higher than that in group B (*P* < 0.05). No considerable differences in PPTs at the other acupoints were observed between the two groups (*P* > 0.05; Table 4).

### 3.4. Correlation among Anxiety, Depression, and PPTs

In the group A, there were no remarkable correlation between the SAS, SDS scores, and PPTs of all the acupoints (*P* > 0.05). However, in the total sample size, SAS scores positively correlated with PPTs of ST29 (R), SP10 (R), SP9 (L), ST36 (R), and LR3 (L) (*P* < 0.05). No remarkable correlation was observed between the SAS scores and PPTs of ST28 (R) and SP9 (R). For SDS, the results of the correlation were similar for those in the group A (*P* > 0.05; Table 5).

## 4. Discussion

This study indicated that 73 (49.7%) out of 147 CPID patients had emotional disorders, and previous study also found that 37 (51%) out of 72 patients with pelvic pain were clinically depressed [33]. Besides, patients with anxiety and depression had a longer course of disease (Table 2). Patients with longer duration of disease usually took medication for longer times. Although we could not rule out that long-term medication treatment had a therapeutic effect on patients' emotions, some patients with a longer course of illness did not experience anxiety and depression. Overall, patients with a longer course of illness are more likely to experience anxiety and depression. Further research can analyze the impact of medication on anxiety and depression in patients with CPID. Some other studies also have reported an association between psychological morbidity and worsening of disease course [34, 35].

Anxiety and depression are common comorbidities in patients with inflammatory diseases [36–38], and inflammation may induce anxiety and depression by directly affecting the neuronal environment through cytokines, or indirectly affecting the neurotransmitter or hormone [39, 40]. In addition, patients with anxiety and depression had higher pain scores and pain can also cause anxiety and depression [41, 42]. CPID is manifested by inflammation and chronic pain; the patients are vulnerable to be accompanied by mood disorders. Several other studies have confirmed higher rates of psychiatric comorbidity (e.g., anxiety and depression) in persons with chronic pain and inflammation [43, 44].

Central and local peripheral sensitization occurs when the body experiences inflammation or pain, causing patients to become more sensitive to pain stimulation. Local hyperalgesia may be caused by peripheral nervous system changes due to prolonged inflammatory processes, and hyperalgesia remote to the area of pain indicate the involvement of the central nervous system. These central changes are associated with ongoing pathological neuronal signals from the painful area [45]. Therefore, we chose the acupoints at the lower abdominal and acupoints of the lower limbs far away from the pain area as the detection points, to entirely explore the pain sensitization.

PPT is a widely used method for measuring pain sensitization. Numerous studies have indicated that patients who experienced chronic pain had significantly lower PPTs compared with healthy controls [46–48]. In addition, the scores of anxiety and depression scales were significantly correlated with PPT [49–51]. Although all patients in this study did not take anxiety-depression psychotropic drugs, we could not determine whether the patient's anxiety and depression were present before being diagnosed with CPID. From the results of this study, there were differences in PPT of some acupoints between the two groups. It could be seen that there was a certain correlation between anxiety and depression and PPT, but the specific causal relationship was not clear.

PPT is also used to measure acupoint sensitization; traditional Chinese medicine believes that the state of acupoints is dynamic and PPT of acupoints changes with the disease. For instance, some studies indicated that lower PPT was observed at the sensitized acupoints in the patients with disease [52, 53], and the underlying mechanism of the acupoint sensitization may be related to the high expression of local allergic substances and nociceptive neuropeptides in the local skin [54]. However, in our study, group A had higher PPT compared with group B in some acupoints. The possible reason for this phenomenon is that the tested acupoints are not completely coincide with the patient's pain area, and these acupoints may have different physical and chemical reaction. Further study is needed to elucidate the difference and potential mechanism in PPT between acupoints and pain areas.

In the current study, the PPT of six acupoints including ST28, ST29, ST36, SP10, SP9, and LR3 were different between the two groups. These acupoints belong to the stomach meridian, spleen meridian, and liver meridian, respectively. Traditional Chinese medicine believes that emotion is closely related to the liver, involves the spleen and stomach. Dysfunction of the liver is frequently accompanied by emotional changes such as depression or anxiety. When the liver qi is too strong, it will affect the spleen and stomach. The acupoints of the liver, spleen, and stomach meridians are usually selected when applying acupuncture to treat emotional disorders in clinical, and the results of this study have provided evidence for these acupoint selection principles. Besides, differences in the PPT of the acupoints between the two groups meant that these acupoints were sensitized by emotion. Previous study showed that intervention at sensitive acupoints is effective in relieving disease symptoms [31]. Therefore, when the CPID patients are accompanied by anxiety or depression, acupuncture at these six acupoints may achieve therapeutic effect to regulate the emotion.

As for the correlation analysis, in the group A, there were no remarkable correlation between the SAS, SDS scores, and PPTs of all the acupoints. But in the total sample size, SAS scores positively correlated with PPTs of ST29 (R), SP10 (R), SP9 (L), ST36 (R), and LR3 (L). The discrepancy in findings may be attributed to sample size and the degree of emotional disorder. When group A was analyzed separately, the sample size was too small to reflect the results of correlation. In a study with 34 juvenile fibromyalgia patients and 31 controls, no relationships were observed between anxiety and pressure pain [46]. Another study with 32 women with frequent episodic tension-type headache and 32 matched healthy women showed that no significant effect of anxiety and depressive levels on PPTs was found [55]. But in another previous research, the correlation between anxiety and PPT was displayed when the sample size was large enough [56]. Besides, there may be differences in PPT due to different levels of anxiety and depression, so there is necessary to increase the sample size and conduct stratified analysis of PPT under different levels of emotional disorders to clarify the correlation between PPT and anxiety or depression in future study. The present results can only reflect the objective data of the samples in this study.

In summary, anxiety and depression should be actively concerned in patients with CPID and treated appropriately. If treatment is not timely, it may become an obstacle to alleviation of the pain. Anxiety and depression affected the PPT of some acupoints in CPID patients, which may cause pain sensitization. Acupuncture at ST28, ST29, ST36, SP10, SP9, and LR3 may be an appropriate treatment for anxiety and depression in patients with CPID.

There were some limitations in this study. First, only the acupoints were detected, the PPT of pain points remains to be further explored. Second, the sample size was relatively small. Third, different anatomical locations may have different sensations; only one method of PPT measurements was applied in this study, and other instruments may be used in different areas to measure the pain sensitization for more accurate results in the future.

## 5. Conclusion

The findings of this study suggest that anxiety and depression can affect the PPT of some acupoints in CPID patients, and these acupoints may be used as stimulation points for acupuncture to treat CPID with emotional disorders.

## Figures and Tables

**Table 1:** The location of acupoints in this study.

Acupoints	Location
Daimai (GB26)	On the lateral abdomen, inferior to the free extremity of the 11th rib, at the same level as the center of the umbilicus
Tianshu (ST25)	On the upper abdomen, at the same level as the center of the umbilicus, 2 cun lateral to the anterior median line
Qihai (CV6)	On the lower abdomen, 1.5 cun inferior to the center of the umbilicus, on the anterior median line
Guanyuan (CV4)	On the lower abdomen, 3 cun inferior to the center of the umbilicus, on the anterior median line
Zhongji (CV3)	On the lower abdomen, 4 cun inferior to the center of the umbilicus, on the anterior median line
Qugu (CV2)	On the lower abdomen, superior to the pubic symphysis, on the anterior median line
Shuidao (ST28)	On the lower abdomen, 3 cun inferior to the center of the umbilicus, 2 cun lateral to the anterior median line
Guilai (ST29)	On the lower abdomen, 4 cun inferior to the center of the umbilicus, 2 cun lateral to the anterior median line
Qichong (ST30)	In the groin region, at the same level as the superior border of the pubic symphysis, 2 cun lateral to the anterior median line, over the femoral artery
Zigong (EX-CA1)	On the lower abdomen, 4 cun inferior to the center of the umbilicus, 3 cun lateral to the anterior median line
Xuehai (SP10)	On the anteromedial aspect of the thigh, 2 cun superior to the medial end of the base of the patella, on the bulge of the vastus medialis muscle
Yinglingquan (SP9)	On the tibial aspect of the leg, in the depression between the inferior border of the medial condyle of the tibia and the medial border of the tibia
Zusanli (ST36)	On the anterior aspect of the leg, 3 cun inferior to ST35, on the line connecting ST35 with ST41
Sanyinjiao (SP6)	On the tibial aspect of the leg, posterior to the medial border of the tibia, 3 cun superior to the prominence of the medial malleolus
Taichong (LR3)	On the dorsum of the foot, between the first and second metatarsal bones, in the depression distal to the junction of the bases of the two bones, over the dorsalis pedis artery

**Table 2:** The demographic data in two groups.

Items	Group A (*n* = 73)	Group B (*n* = 74)	Statistical value	*P* value
Age (years)	30.00 (26.00–36.50)	30.50 (27.00–36.00)	−0.246	0.805
BMI	20.40 (18.73–22.60)	20.16 (18.83–22.42)	−0.143	0.886
Duration of disease (months)	12.00 (7.00–36.00)	7.00 (4.00–24.00)	−2.637	0.008^*∗*^
Educational level, *n* (%)			0.695	0.706
Primary education or less	3 (4.1%)	2 (2.7%)		
Secondary education	18 (24.7%)	15 (20.3%)		
Tertiary education	52 (71.2%)	57 (77.0%)		
Resting blood pressure (mm Hg)				
Systolic	114.00 (112.00–120.00)	116.00 (108.00–120.00)	−0.246	0.806
Diastolic	76.00 (70.00–79.00)	74.00 (70.00–78.00)	−1.228	0.220

BMI: body mass index (calculated as weight in kilograms divided by height in meters squared). ^*∗*^, *P* < 0.05.

**Table 3:** Differences in the emotion and pain scores between the two groups.

Items	Group A	Group B	Statistical value	*P* value
SAS	49.00 (45.00–54.00)	38.50 (34.00–41.00)	−9.151	≤0.001^*∗*^
SDS	53.00 (46.00–59.00)	40.00 (35.00–43.00)	−7.721	≤0.001^*∗*^
VAS	5.15 ± 1.82	4.20 ± 1.93	3.083	0.002^*∗*^
SF-MPQ	14.30 (10.70–19.00)	10.25 (8.38–14.70)	−3.960	≤0.001^*∗*^

SAS: Self-Rating Anxiety Scale; SDS: Self-Rating Depression Scale; VAS: Visual Analog Scale; SF-MPQ: short-form McGill Pain Questionnaire. ^*∗*^, *P* < 0.05.

**Table 4:** Differences in the PPT between the two groups.

Acupoints	Group A	Group B	Statistical value	*P* value
GB26 (R)	62.85 (42.68–100.10)	50.63 (39.46–85.26)	−1.538	0.124
GB26 (L)	63.65 (41.28–87.58)	51.25 (37.05–77.43)	−1.635	0.102
ST25 (R)	65.75 (43.70–104.35)	54.58 (37.78–77.85)	−1.753	0.080
ST25 (L)	59.10 (45.25–102.80)	53.10 (42.53–72.53)	−1.623	0.105
CV6	62.30 (37.23–88.80)	53.00 (38.89–72.13)	−1.189	0.234
CV4	57.55 (39.63–89.78)	48.75 (37.26–82.41)	−1.383	0.167
CV3	55.00 (43.20–85.10)	49.58 (35.63–74.51)	−1.257	0.209
CV2	55.05 (40.25–89.25)	48.93 (37.90–84.45)	−1.360	0.174
ST28 (R)	57.05 (41.85–89.95)	46.65 (35.36–66.60)	−2.086	**0.037** ^*∗*^
ST28 (L)	59.70 (43.23–90.98)	48.95 (37.40–84.91)	−1.387	0.165
ST29 (R)	59.70 (42.18–88.25)	47.05 (33.59–70.80)	−2.110	**0.035** ^*∗*^
ST29 (L)	54.85 (39.23–83.05)	48.78 (36.29–77.26)	−1.273	0.203
ST30 (R)	58.85 (39.25–87.13)	47.48 (34.51–73.35)	−1.730	0.084
ST30 (L)	59.35 (41.53–87.00)	48.50 (35.58–73.76)	−1.759	0.079
EX-CA1 (R)	57.20 (41.38–86.78)	48.48 (37.23–73.44)	−1.399	0.162
EX-CA1 (L)	60.95 (42.20–77.95)	49.50 (37.15–81.25)	−1.147	0.251
SP10 (R)	69.25 (50.95–92.93)	54.18 (38.98–80.01)	−2.737	**0.006** ^*∗*^
SP10 (L)	69.70 (44.18–93.33)	57.48 (40.30–92.90)	−1.368	0.171
SP9 (R)	70.40 (45.73–100.08)	52.95 (38.69–82.05)	−2.129	**0.033** ^*∗*^
SP9 (L)	69.50 (47.13–98.95)	52.63 (40.15–83.53)	−2.073	**0.038** ^*∗*^
ST36 (R)	77.40 (46.93–114.53)	55.93 (42.48–82.39)	−2.075	**0.038** ^*∗*^
ST36 (L)	68.60 (48.23–101.73)	58.88 (45.05–94.25)	−1.339	0.181
SP6 (R)	61.70 (41.63–93.40)	53.20 (39.83–81.79)	−1.447	0.148
SP6 (L)	58.80 (41.10–97.38)	50.63 (35.03–74.69)	−1.518	0.129
LR3 (R)	64.35 (43.98–101.40)	53.75 (39.44–81.53)	−1.893	0.058
LR3 (L)	66.30 (47.73–98.78)	51.00 (38.81–76.90)	−2.082	**0.037** ^*∗*^

PPT: pressure pain threshold; R: right; L: left. ^*∗*^, *P* < 0.05. The bold values indicate significant statistical values between the two groups.

**Table 5:** Correlation among anxiety, depression, and PPT in CPID patients.

			ST28 (R)	ST29 (R)	SP10 (R)	ST36 (R)	SP9 (R)	SP9 (L)	LR3 (L)
Group A (*n* = 73)	SAS	Spearman	0.049	0.118	0.157	0.030	−0.005	0.043	0.058
		*P* value	0.683	0.320	0.184	0.803	0.996	0.718	0.625
	SDS	Spearman	0.037	0.141	−0.001	0.051	−0.001	0.108	0.090
		*P* value	0.756	0.233	0.096	0.670	0.995	0.362	0.451

Group A + group B (*n* = 147)	SAS	Spearman	0.162	0.191	0.231	0.178	0.148	0.169	0.177
		*P* value	0.050	**0.020** ^*∗*^	**0.005** ^*∗*^	**0.031** ^*∗*^	0.073	**0.041** ^*∗*^	**0.032** ^*∗*^
	SDS	Spearman	0.101	0.123	0.144	0.124	0.076	0.129	0.149
		*P* value	0.224	0.139	0.082	0.135	0.359	0.119	0.072

^*∗*^, *P* < 0.05. The bold values indicate significant statistical values between the two groups.

## Data Availability

The datasets are available from the corresponding author on reasonable request.
